# Radiographers’ workload and burnout on performance: an empirical study

**DOI:** 10.3389/fpubh.2024.1419784

**Published:** 2024-08-13

**Authors:** Wejdan M. Arif

**Affiliations:** Department of Radiological Sciences, College of Applied Medical Sciences, King Saud University, Riyadh, Saudi Arabia

**Keywords:** radiographers, radiology, burnout, stress, anxiety, work-life, well-being, workload

## Abstract

**Study purpose:**

To assess the prevalence of burnout among radiographers, and whether demographic variables and work-related factors had any influence on burnout and perceived stress among them.

**Methods:**

A cross-sectional quantitative survey design is adopted in this study. The participants included radiographers from Saudi Arabia. Both Maslach Burnout Inventory (MBI) and Perceived Stress Scale (PSS) were used for data collection. Participation was voluntary, and the survey was conducted online, resulting in 322 final responses considered for the data analysis.

**Results:**

The mean emotional exhaustion (EE) score achieved was 26.01, representing medium burnout risk. However, the mean depersonalization (DP: *μ* = 25.25) and personal accomplishment (PA: *μ* = 23.65) represented high burnout risk among radiographers. Statistically significant differences (*p* < 0.05) were observed among the participants grouped by genders, age groups, nature of work type, and work experience. The mean perceived stress score for radiographers was identified to be 27.8, indicating high.

**Conclusion:**

The findings underscore the critical need for targeted interventions and support mechanisms within the radiology profession, particularly focusing on younger radiographers and those with extensive work experience.

## Introduction

The concept of burnout was initially introduced by Freudenberger in 1974 and is currently defined as a syndrome that encompasses emotional exhaustion, depersonalization, and decreasing personal accomplishment (1). The 11th Revision of the International Classification of Diseases (ICD-11) incorporates burnout as an occupational condition (2), and provides a description of many factors that influence individuals’ health status or their engagement with health services. Burnout is a phenomenon that arises because of perceived stress in work environments, leading individuals to experience feelings of apathy, cynicism, indifference, and detachment from their surroundings. In specific instances of high significance, burnout has the potential to cause severe psychopathological harm, leading to various negative outcomes such as insomnia, difficulties within marital or familial relationships, escalated substance abuse, and increased absenteeism. Consequently, these consequences may potentially compromise the quality of care or service delivered by the individuals involved (3).

Burnout, a pervasive occupational phenomenon, casts a shadow over the professional landscape of radiographers, influencing both their work life and overall well-being (4, 5). Radiographic work is unique due to its high cognitive load, the necessity for sustained attention to detail, and exposure to radiation, all of which contribute significantly to burnout. These factors, coupled with the demanding nature of the job, amplify the risk of emotional exhaustion, depersonalization, and reduced personal accomplishment.

Recent statistics highlight the alarming prevalence of burnout among healthcare professionals, including radiographers. Studies indicate that burnout rates among radiographers range from 30 to 70%, underscoring the critical need to address this issue (6–8). Emotional exhaustion, defined as feelings of being emotionally overextended and depleted of emotional resources due to prolonged stress or excessive demands in one’s work or personal life, is particularly prevalent (7). This emotional weariness can translate into decreased job performance, compromising the quality and accuracy of diagnostic imaging (6). In a field where precision is paramount, the consequences of a fatigued mind can be significant, potentially leading to misinterpretations and diagnostic errors.

Depersonalization, characterized by a negative, detached, and cynical attitude toward one’s job and the people involved in it, introduces a sense of detachment and cynicism in the radiographer-patient relationship (9). The empathetic connection that is pivotal in healthcare can be strained, with depersonalized radiographers potentially exhibiting a diminished capacity to understand and respond to the emotional needs of patients (10). Communication breakdowns may ensue, impacting the overall patient experience and potentially hindering the establishment of trust, which is integral in the medical field (11).

Moreover, the erosion of personal accomplishment, which focuses on feelings of competence and successful achievement in one’s work, can have a profound impact on job satisfaction and motivation among radiographers (11). As they face increasing challenges and stressors, the diminished sense of achievement may lead to a pervasive feeling of ineffectiveness. This can create a negative feedback loop where a lack of fulfillment contributes to reduced enthusiasm for the job, further exacerbating burnout (12).

Beyond the professional sphere, the repercussions of burnout extend into the personal lives of radiographers. The chronic stress associated with burnout can manifest physically and mentally, contributing to fatigue, insomnia, and even more severe health issues (11). The toll on mental health is particularly noteworthy, with burnout increasing the risk of anxiety and depression among radiographers. These personal struggles not only affect the well-being of the individual but also have the potential to spill over into their professional lives, creating a cycle of stress and burnout that is challenging to break.

Addressing burnout in radiographers is imperative for the sustainability of healthcare systems (12, 13). Strategies to mitigate burnout must encompass organizational interventions, such as creating supportive work environments, implementing workload management measures, and fostering a culture that prioritizes employee well-being. Providing resources for stress management, counseling, and promoting a healthy work-life balance are pivotal components of these efforts (14, 15).

The effects of burnout on radiographers are far-reaching and multifaceted, influencing both their professional capabilities and personal lives (8, 13). Recognizing and addressing burnout is not only crucial for the individual radiographer’s well-being but also for maintaining the high standards of patient care and safety within the radiology profession (16–18). As the healthcare landscape continues to evolve, prioritizing the mental and emotional health of radiographers becomes not only an ethical imperative but a strategic necessity for building resilient and sustainable healthcare systems (19–21).

The burnout prevalence among the radiologists varied across the regions, typically ranging between 33 to 88% (22). There is a paucity of comprehensive data on the frequency of burnout among healthcare personnel in different healthcare sectors in Middle Eastern countries (23–25). Roughly 33% of medical practitioners experience burnout, which can have negative consequences on both their own well-being and the quality of treatment they provide (26). The problem of burnout has become a serious and formidable challenge in the field of public health. Regrettably, there is a lack of comprehensive understanding regarding the ailment, and the acceptance of its diagnosis is infrequent (27). Burnout can lead to fatigue-induced changes in pain perception, compromised cardiovascular health, depression, and musculoskeletal discomfort, all of which have adverse medical and psychological consequences.

The collective occurrence of complete burnout among area physicians is 24.5%. The sub-components of burnout have been assessed to have a high pooled prevalence of 44.26% for emotional exhaustion (EE), 37.83% for depersonalization (DP), and 36.57% for low personal accomplishment (PA) (5). Healthcare organizations experience substantial effects from burnout among health professionals, including heightened rates of absenteeism and error likelihood, frequent work delays, decreased productivity, job dissatisfaction, conflicts between and within professions, high turnover and resignation rates, and a perceived decline in the quality of care by users. In a recent study conducted in Saudi Arabia (28), high overall burnout was reported by 24.1% of respondents, high emotional exhaustion (EE) by 56.5%, high depersonalization by 31.5%, and low sense of personal accomplishment (PA) by 64.8%.

Therefore, the aim of this study is to assess the prevalence of burnout among radiographers, and whether demographic variables and work-related factors had any influence on burnout and perceived stress among them.

### Research questions and objectives

The primary objective of this study is to assess the prevalence of burnout among radiographers in Saudi Arabia. Specifically, the research aims to address the following questions:

1. What is the prevalence of burnout among radiographers in Saudi Arabia?
2. How do demographic variables (such as gender, age, and work experience) influence burnout levels among radiographers?
3. How do work-related factors (such as nature of work and presence of training courses on managing emotional factors) impact burnout and perceived stress among radiographers?

## Methods

A cross-sectional quantitative survey design is adopted in this study for achieving the above specified aim.

### Study settings and participants

The participants included radiographers from Saudi Arabia. As the study was focused specifically on radiographers, a, purposive sampling technique (28) was adopted in this study, recruiting only radiographers who are currently working across Saudi Arabia. Participants for this study were recruited through a combination of online and social media channels. Specifically, all radiographers across all medical centers in the radiology department in Saudi Arabia, were targeted. These radiographers were sent an email inviting them to complete an online questionnaire designed to assess burnout and perceived stress. The email contained detailed information about the study’s purpose, the voluntary nature of participation, and assurances of confidentiality and anonymity. Additionally, reminders were sent to encourage participation and ensure a robust response rate. The survey was online for a period of 5 weeks.

### Questionnaire design

The first part of the survey comprised demographic and work-related questions, addressing gender, age range, nature of work, extent of experience in a radiotherapy department, presence of training courses on managing emotional factors, and psychological support.

The second part of the survey was based on the Maslach Burnout Inventory (MBI) questionnaire (29, 30). The MBI is the most widely used tool to evaluate burnout in healthcare workers. It assesses three different dimensions: emotional exhaustion (EE), depersonalization (DP) and personal accomplishment (PA). The MBI survey is composed by 22 items and it is divided in 3 subscales: EE (9 items), DP (5 items), and PA (8 items). For each item the MBI uses a 7-point response scale, whose extremes are “never” and “every day.” Scores within individual burnout domains can either be used as continuous variables or categorized into indicators of low, medium, and high risk of burnout using established cut-offs (Table 1). It is important to note that high levels in EE and DP subscales are associated to high burnout, while high levels in PA subscale are associated to low burnout. The MBI has been extensively validated across various occupational groups, including healthcare professionals (32–34), demonstrating high reliability and validity. Its use in this study ensures that the measurement of burnout is both accurate and comparable to other studies in the field.

**Table 1:** Reference value of MBI and PSS questionnaire scores (30, 31).

		Low	Medium	High
MBI	EE	<=17	18–29	>=30
	DP	<=5	6–11	>=12
	PA	<=33	34–39	>=40
PSS	PS	0–13	14–26	27–40

The third part of the survey includes the Perceived Stress Scale (PSS) with the 10 questions proposed by Cohen et al. (35), which can be scored from 0 to 4 (0: never; 4: very often). This scale is used to assess everyone’s perception of situations of daily life, and their reaction in response to such events. Perceived stress was assessed using the Perceived Stress Scale (PSS), a tool designed to measure the perception of stress. The PSS is known for its reliability and validity, making it a suitable instrument for this study. It provides a measure of the degree to which situations in one’s life are appraised as stressful, and its psychometric properties have been validated in numerous studies. The use of the PSS allows for a reliable assessment of perceived stress levels among the participants, complementing the burnout measurements obtained through the MBI. PSS was chosen because it is widely used to determine how unpredictable, uncontrollable, and overloaded respondents find their lives; consistent with other studies (35).

### Data collection

All the participants were fully informed about the study through an information sheet attached with the online survey. An informed consent was taken from all the participants using a check button, before starting the survey. The participation was voluntary and the participants were assured of their anonymity and their rights with respect to the data. At the end of 5 weeks study, a total of 356 responses were received, out of which 34 responses were incomplete, and were removed, resulting in 322 responses, which were used in data analysis.

### Data analysis

To attain the objectives of the research, the following statistical methods were used for data analysis:

1. Descriptive statistics: used to characterize the participants’ demographic data, including frequencies, means, and standard deviations.
2. Two-sample *t*-test with unequal variances: employed to compare differences in burnout and perceived stress scores between male and female participants.
3. Analysis of variance (ANOVA): used to compare differences in burnout and perceived stress scores across different age groups, nature of work (full-time vs. part-time), and work experience levels.
4. Correlation analysis: Pearson correlation coefficients were calculated to examine the relationships between burnout dimensions (EE, DP, PA) and perceived stress (PS).

The statistical analysis was performed using the Statistical Package for the Social Sciences (SPSS) IBM Version 24. All statistical tests were two-tailed, and a *p*-value of less than 0.05 was considered statistically significant.

### Ethical procedure

The study received approval from the Permanent Committee for Scientific Research Ethics at King Saud University. The data collection and analysis procedure were carried out in compliance with all relevant ethical norms. To ensure confidentiality and anonymity, several measures were implemented. The email invitation included a link to an online consent form that provided detailed information about the study’s purpose, procedures, and the voluntary nature of participation. Participants were required to read and acknowledge the consent form before accessing the questionnaire. Data collection was conducted anonymously; no personally identifiable information was collected, and responses were recorded without any linkage to the participants’ identities. Additionally, the online platform used for the questionnaire was secured to protect data privacy, and only the research team had access to the anonymized data.

## Results

Table 2 presents the participants demographics. Participants were appropriately distributed across both genders with females representing 41.9% and males representing 58.1%. Almost 80% of the participants were aged between 18 and 40 years. About one-third of the participants were employed full-time. Majority of the participants has work experience between 4 and 6 years (43.5%), followed by 0–3 years (32.6%), 7–9 years (14.3%), and above 9 years (9.6%).

**Table 2:** Participants demographics.

		*N*	Relative frequency
Gender	Male	187	58.1%
	Female	135	41.9%
Age (in years)	18–30	169	52.5%
	31–40	95	29.5%
	41–50	52	16.1%
	>=51	6	1.9%
Nature of work	Full time	242	75.2%
	Part time	80	24.8%
Work experience (in years)	0–3	105	32.6%
	4–6	140	43.5%
	7–9	46	14.3%
	>=10	31	9.6%

In relation to the training courses in managing emotional factors, 56.2% participants stated they receive training while 41.9% stated that they do not, and the remaining stated that they do not know. About 50% of the participants (51.3%) stated that they receive psychological support. The mean EE score achieved was 26.01, representing medium burnout risk. However, the mean DP (μ = 25.25) and PA (μ = 23.65) represented high burnout risk among radiographers.

### Burnout scores

Table 3 presents a detailed analysis of burnout scales among radiographers based on various demographic and professional factors. The data suggests that there are significant differences in burnout levels among different groups. Firstly, in terms of gender, male radiographers exhibit lower EE (*μ* = 25.3 vs. *μ* = 26.9, *p* = 0.0342) and DP (*μ* = 24.6 vs. *μ* = 26.1, *p* = 0.0263) compared to their female counterparts. However, there is no significant difference in PA between genders. Secondly, when considering age, radiographers aged 18–30 experience higher EE (*μ* = 26.9, *p* = 0.0138) and DP (*μ* = 26.3, *p* = 0.002) compared to those in other age groups. PA shows a marginal difference across age groups (*p* = 0.0504). Thirdly, the nature of work significantly influences burnout.

**Table 3:** Differences in burnout sub-scales among the participants groups.

Variables		N	EE			DP			PA		
			Mean	SD	*p*-value	Mean	SD	*p*-value	Mean	SD	*p*-value
Gender	Male	187	25.3	73.4	0.0342*	24.6	56.3	0.0263	23.5	33.4	0.3642
	Female	135	26.9	62		26.1	41.1		23.7	29.1	
Age (in years)	18–30	169	26.9	65.1	0.0138*	26.3	49.5	0.002*	23.4	31.5	0.0504
	31–40	95	23.7	84.4		22.9	56.4		23.6	26.8	
	41–50	52	27.2	47.6		26.2	30.4		25.2	38.3	
	>=51	6	28.0	32.8		25.7	51.1		19.5	25.9	
Nature of work	Full time	242	29.9	18	<0.0001*	28.3	19.4	<0.0001*	23.7	31.5	0.2281
	Part time	80	14.2	38.5		15.9	27.4		23.2	31.8	
Work experience (in years)	0–3	105	26.6	70.8	0.002*	25.7	49.9	0.0007*	22.9	31.7	0.2633
	4–6	140	24.4	77.9		23.9	58.3		23.7	29.1	
	7–9	46	29.6	24.5		28.7	17.4		24.2	30.2	
	>=10	31	26.2	63.7		24.9	41.7		25.0	43.7	

SD, standard deviation; df, degrees of freedom; *Statistically significant difference.

Full-time radiographers demonstrate higher EE (*μ* = 29.9, *p* < 0.0001) and DP (*μ* = 28.3, *p* < 0.0001) compared to part-time colleagues. However, there is no significant difference in PA between the two groups. Lastly, work experience plays a role in burnout. Radiographers with 0–3 years of experience exhibit higher EE (*μ* = 26.6, *p* = 0.002) and DP (*μ* = 25.7, *p* = 0.0007) compared to those with more experience. There is no significant difference in PA across different experience levels.

### Perceived stress scores

The mean PS scale score for radiographers was identified to be 27.8, indicating high PS. Table 4 provides insights into the PS levels among radiographers, considering various demographic and professional variables. The data indicates that there is no statistically significant difference in PS between male and female radiographers (*μ* = 27.5 vs. *μ* = 28.1, *p* = 0.165). Both genders seem to experience comparable levels of PS. While there is no overall significant difference in PS across different age groups (*p* = 0.0965), there is a noteworthy observation. Radiographers aged 41–50 appear to have higher PS (*μ* = 28.8) compared to other age brackets, although this difference does not reach statistical significance. The nature of work emerges as a significant factor influencing PS levels. Full-time radiographers report significantly higher levels of stress (*μ* = 29.1, *p* < 0.0001) compared to their part-time counterparts. This suggests that the demands and workload associated with full-time positions contribute to elevated stress levels. Although there is no statistically significant difference in PS across various experience levels (*p* = 0.0669), there are trends worth exploring. Radiographers with 7–9 years of experience stand out with the highest PS (*μ* = 29.4), followed closely by those with over 10 years of experience. While not statistically significant, these trends may indicate a potential relationship between work experience and PS (Table 5).

**Table 4:** Differences in burnout sub-scales among the participants groups.

Variables		*N*	*PS*		
			Mean	SD	*p*-value
Gender	Male	187	27.5	31.7	0.165
	Female	135	28.1	34.9	
Age (in years)	18–30	169	28.0	28.9	0.0965
	31–40	95	26.7	45.0	
	41–50	52	28.8	22.6	
	>=51	6	29.8	35.4	
Nature of work	Full time	242	29.1	25.2	<0.0001*
	Part time	80	23.9	37.6	
Work experience (in years)	0–3	105	27.7	26.9	0.0669
	4–6	140	27.1	41.8	
	7–9	46	29.4	25.0	
	>=10	31	29.1	21.5	

SD, standard deviation; df, degrees of freedom; *Statistically significant difference.

**Table 5:** Correlation matrix between burnout and PS sub scales.

	EE	DP	PA	PS
EE	1			
DP	0.720785	1		
PA	0.08272	0.0798	1	
PS	0.309686	0.422581	0.044541	1

The correlation matrix reveals significant relationships among the key variables measured in this study—EE, DP, PA, and PS. EE and DP exhibit a strong positive correlation (*r* = 0.7208), indicating that as radiographers experience higher EE, they also tend to report higher levels of DP. Additionally, both EE and DP show moderate positive correlations with PS (*r* = 0.3097 and *r* = 0.4226, respectively), suggesting that increased EE and DP are associated with elevated levels of PS. The weak positive correlation between EE and PA (*r* = 0.0827) hints at a nuanced relationship where higher EE may be marginally linked to increased PA. Overall, these correlations underscore the interconnected nature of burnout components and PS, providing valuable insights into the complex dynamics within the radiology profession.

## Discussion

This study has explored occupational burnout among radiographers in Saudi Arabia using MBI and PSS subscales. The results from the study indicated that radiographers exhibit moderate levels of burnout with respect to EE and high levels of burnout with respect to DP and PA. In addition, high levels of stress were identified among the radiographers. Previous studies have focused on assessing burnout using MBI scales on various healthcare personnel (36–39), and on radiographers (8, 10–21). Most of the previous research in this area has indicated that burnout had negative effects not only on performance but also on individual well-being (10–15).

Within the study, radiographers demonstrated a notably moderate median score for EE (*μ* = 26.01) and high median scores for DP (*μ* = 25.25) and PA (*μ* = 23.65). However, the median score for EE was found to be close to the reference value for high risk. Additionally, a substantial proportion of respondents (40.9, 95.3, and 96.2% respectively) exceeded the established thresholds for EE, DP, and PA. The findings in this study reflected a higher burnout burden compared to the studies in other regions (10–14, 30) and in Saudi Arabia (8), indicating high levels of burnout among the radiographers in Saudi Arabia compared to other regions.

The high levels of burnout among radiographers in Saudi Arabia can be attributed to several specific factors. The rise in chronic illnesses like cancer and heart diseases in Saudi Arabia (40–43), coupled with the after-effects of COVID-19 (44–46), has led to an increased inflow of patients, significantly increasing the workload for radiographers. This heightened workload not only demands more time and effort but also brings considerable emotional strain from patient care, as radiographers often deal with critically ill patients and complex cases, leading to higher levels of stress and emotional exhaustion.

It can be observed from the findings that female radiographers are experiencing higher burnout compared to male radiographers, especially in relation to EE and DP. Societal and workplace factors may contribute to this disparity. In many societies, including Saudi Arabia, women often face additional pressures from balancing professional responsibilities with traditional roles in the household, leading to greater stress and burnout. Workplace dynamics, such as potential gender biases and limited support systems, may also exacerbate the burnout experienced by female radiographers.

Furthermore, younger radiographers (18–30 years) and older radiographers (>41 years) were found to be experiencing higher burnout levels compared to middle-aged radiographers (31–40 years). Younger radiographers may face burnout due to the challenges of adjusting to the demanding nature of the profession and developing coping mechanisms early in their careers. On the other hand, older radiographers may experience burnout due to cumulative stress over the years and possible declines in physical stamina and resilience. These findings are consistent with previous studies indicating that experience levels can influence burnout rates (47, 48).

Interestingly, part-time radiographers experienced less burnout compared to full-time radiographers, indicating the negative impact of longer shifts on radiographers’ work-life balance and the need for appropriate working hours (49, 50). The perceived stress among part-time radiographers is significantly less compared to full-time radiographers, suggesting that reduced hours can alleviate some of the stress and burnout. It is also noteworthy that there were no statistically significant differences among the participants grouped by gender, age, and experience in relation to perceived stress, indicating that stress levels are similarly experienced among all participant groups.

The high positive correlation between EE and DP suggests that as radiographers experience higher levels of EE, there is a strong tendency for them to also exhibit higher levels of DP. This implies that burnout, particularly in terms of emotional exhaustion, may contribute significantly to feelings of detachment or cynicism in interpersonal relationships with patients and colleagues. Reducing emotional exhaustion could therefore help decrease perceived stress and improve radiographers’ relationships with patients, fostering a more supportive and empathetic care environment. The positive correlation between EE and PS indicates that as EE increases, radiographers are more likely to report higher levels of PS. Addressing EE may contribute to reducing PS among radiographers. The moderate positive correlation between DP and PS implies that higher levels of DP are associated with increased PS. This suggests that addressing DP, along with EE, may contribute to mitigating overall stress levels among radiographers.

The findings of this study on radiographers’ workload, burnout, and performance carry both practical and theoretical implications. Practically, the results highlight the urgent need for interventions and support mechanisms within the radiology profession, particularly in Saudi Arabia, to address the high levels of burnout identified among radiographers. Examples of specific interventions include implementing workload management strategies, such as optimizing shift schedules and reducing overtime; providing emotional support through counseling services and peer support groups; and fostering a healthy work environment by promoting a culture of recognition and appreciation. Additionally, creating policies that support work-life balance, such as flexible working hours and part-time options, can be instrumental in mitigating burnout and improving overall well-being.

The observed differences in burnout levels based on gender, age, nature of work, and work experience underscore the importance of targeted interventions tailored to specific demographic and professional groups. For instance, recognizing the heightened burnout risk among younger radiographers and implementing measures to support their well-being early in their careers can be crucial. Furthermore, developing gender-sensitive policies that address the unique challenges faced by female radiographers can help reduce their burnout levels.

Theoretically, this study contributes to the growing body of research on burnout in healthcare professionals, specifically radiographers. The inclusion of perceived stress as a key variable adds depth to the understanding of the psychological aspects of burnout. The study aligns with existing literature by confirming the substantial impact of burnout on radiographers’ well-being and job performance. The use of established tools such as the MBI and PSS enhances the reliability and comparability of the findings with broader research in the field. The identification of demographic and professional factors influencing burnout provides theoretical insights into the nuanced dynamics of burnout within the context of radiography.

While this study offers valuable insights into the burnout levels among radiographers in Saudi Arabia, it is not without limitations. Firstly, the cross-sectional design restricts the establishment of causal relationships between variables, emphasizing the need for future longitudinal studies to track the dynamics of burnout over time. The reliance on self-reported measures, such as the Maslach Burnout Inventory and Perceived Stress Scale, introduces the potential for response bias. Future research could incorporate objective measures or explore additional factors contributing to burnout. The study’s focus on Saudi Arabian radiographers may limit the generalizability of findings to other cultural and professional contexts. A broader, multinational approach would enhance the external validity of research in understanding the universality or cultural specificity of burnout experiences. Additionally, while the study identifies associations between burnout, demographic factors, and perceived stress, a deeper exploration of organizational factors and interventions could offer more comprehensive insights. Future research could delve into the effectiveness of specific interventions and strategies in mitigating burnout among radiographers, contributing to the development of targeted and evidence-based approaches for improving well-being in this critical healthcare profession.

## Conclusion

In conclusion, this empirical study sheds light on the prevalent burnout levels among radiographers in Saudi Arabia, emphasizing the multifaceted impact of burnout on both professional performance and overall well-being. The findings underscore the critical need for targeted interventions and support mechanisms within the radiology profession, particularly focusing on younger radiographers and those with extensive work experience. Addressing burnout is not only essential for the well-being of radiographers but also for the overall quality of patient care and the efficiency of healthcare systems. The correlation between burnout components and perceived stress highlights the interconnected nature of these factors, calling for comprehensive strategies that address both dimensions.

The insights gained from this study can inform policy changes and workplace practices in healthcare settings globally. Implementing evidence-based strategies to mitigate burnout can enhance the resilience of radiographers, ultimately contributing to the sustainability and quality of healthcare systems. While the study contributes valuable insights into the dynamics of burnout, it is not without limitations, prompting the call for future research to adopt longitudinal designs, explore objective measures, and extend the investigation to a broader, multinational context. By addressing these limitations and delving into organizational interventions, future research can pave the way for effective solutions to alleviate burnout and improve the professional lives of radiographers, ensuring high standards of patient care and operational efficiency in healthcare systems worldwide.

## Data availability statement

The original contributions presented in the study are included in the article/supplementary material, further inquiries can be directed to the corresponding author.

## Ethics statement

The studies involving humans were approved by Institutional Review Board (IRB) of King Saud University. The studies were conducted in accordance with the local legislation and institutional requirements. The participants provided their written informed consent to participate in this study.

## Author contributions

WA: Conceptualization, Data curation, Formal analysis, Funding acquisition, Investigation, Methodology, Project administration, Resources, Software, Supervision, Validation, Visualization, Writing – original draft, Writing – review & editing.
